# Web-Based Digital Storytelling for Endometriosis and Pain: Qualitative Pilot Study

**DOI:** 10.2196/37549

**Published:** 2023-03-14

**Authors:** A Fuchsia Howard, Heather Noga, Gurkiran Parmar, Lan Kennedy, Sarah Aragones, Roop Bassra, Lauren Gelfer, Edurne Lopez de Arbina, Jessica Sutherland, Catherine Allaire, John L Oliffe, Leanne M Currie, Holly Yager, Paul J Yong

**Affiliations:** 1 School of Nursing The University of British Columbia Vancouver, BC Canada; 2 Women's Health Research Institute British Columbia Women's Hospital & Health Centre Vancouver, BC Canada; 3 Department of Obstetrics and Gynecology The University of British Columbia Vancouver, BC Canada; 4 Endometriosis Patient Advisory Board British Columbia Women's Hospital and Health Centre Vancouver, BC Canada; 5 British Columbia Women's Centre for Pelvic Pain and Endometriosis British Columbia Women's Hospital and Health Centre Vancouver, BC Canada; 6 Department of Nursing University of Melbourne Parkville Australia; 7 Reproductive Health and Fertility Counselling Vancouver, BC Canada

**Keywords:** digital storytelling, endometriosis, pain, chronic pain, painful sex, dyspareunia, integrated knowledge translation, patient-oriented research, community-based participatory research, qualitative research, arts-based research, group therapy, sex therapy

## Abstract

**Background:**

Endometriosis is a complex chronic disease characterized by pain, including painful sex, that can contribute to considerable sexual function, self-esteem, and relationship challenges. Digital storytelling is an arts-based, participatory methodology wherein individuals create and share their illness experiences in detailing their lived experiences.

**Objective:**

The study objective was to pilot-test a web-based digital storytelling workshop focused on endometriosis to understand storytellers’ experiences of workshop participation. We assessed the feasibility of story cocreation and sharing, including the emotional impact of workshop participation, the acceptability of the workshop for the subject matter, and the storytellers’ willingness to share their stories with broader audiences as a method for knowledge translation.

**Methods:**

This study used a community-based participatory methodology supplemented with patient-oriented research and integrated knowledge translation. Study participants, referred to as storytellers, cocreated 3- to 5-minute individual digital stories about their lived experiences of endometriosis during a web-based workshop (comprising five 2-hour sessions over 6 weeks) facilitated by The Center for Digital Storytelling. Data were collected through participant observations at the workshop, storyteller weekly reflective journals, and an end-of-workshop focus group interview with storytellers. These data were analyzed using a qualitative interpretive description approach.

**Results:**

A total of 5 women and 1 nonbinary storyteller aged 19 to 39 years who had experienced endometriosis for 4 to 22 years participated in the study. We characterized storytelling workshop participation and the acceptability of story cocreation by describing participants’ experiences of opportunity, commitment, and connection; complex emotions that were healing; and a desire to share. Feasibility was demonstrated through 100% engagement in the workshops. All 6 storytellers reported feeling empowered by publicly sharing their cocreated digital stories through social media and the *Sex, Pain & Endometriosis* website.

**Conclusions:**

Despite the complexities of the story-building process, the workshop and the cocreation and sharing of digital stories were feasible. The storytellers found that this process allowed for emotional healing and personal empowerment by offering a unique way to talk about painful sex, which also facilitated a connection among those in the workshop. The use of digital storytelling as a knowledge translation tool shows promise, and this approach also has potential as a therapeutic intervention.

## Introduction

### Background

Endometriosis is a chronic, systemic inflammatory condition that affects approximately 190 million people worldwide and is characterized by the presence of endometrium-like tissue outside the uterus [1]. Symptoms can include infertility, fatigue, severe pain, dysmenorrhea (menstrual pain), painful defecation (dyschezia), painful urination (dysuria), and chronic pelvic pain [2]. Over 50% of those with endometriosis also experience pain during sexual activity that negatively affects their quality of life and intimate relationships [2-4]. Sexual health is well recognized as a critical component of overall health [3]. Although endometriosis is common, its effects on sexual health, combined with the social stigma around discussing female sexuality, can create barriers to medical care, support, and counseling [4-6]. Pain that occurs before, during, or after sexual intercourse is a complication of endometriosis that can include superficial dyspareunia (pain at the initial entry into the vagina), deep dyspareunia (pain associated with deep penetration), and musculoskeletal pain (ie, back, hip, and abdomen). When ignored, this can cascade beyond physical pain to result in psychological, sexual, and interpersonal struggles [3-5,7]. Furthermore, the usual cognitive and emotional responses to pain may worsen pain intensity, abrogate sexual satisfaction, cause hypervigilance during sexual activity, and exacerbate the fear of all painful risks [7]. Shame, hopelessness, guilt, loss of self-esteem, and frustration have been associated with symptoms of depression and anxiety [8]. Not only are close, romantic relationships affected, but some people also describe social isolation as a result [3,4].

In clinical research, pain is typically defined by standardized classification scales that ask patients to identify their pain numerically through visual analog scales or describe it verbally in an unfamiliar clinical or research context. However, pain measures do not necessarily capture the breadth and depth of the experience of living with chronic pain and, therefore, may not be relatable to a patient. Web-based tools have shown promise in conveying health information to facilitate help seeking, reduce emotions of isolation and foster a sense of connection, and validate experiences and mitigate fears of stigma [9-14].

Our team of patients, clinicians, and researchers codeveloped the evidence-based website *Sex, Pain & Endometriosis* [15] to assist people in understanding their endometriosis-associated painful sex. During website development, patient stakeholders expressed a need to engage media components to reduce emotions of isolation, combat stigma, and assist people in finding validation. Storytelling has been suggested as one of the most powerful aspects of engagement [10,11], with digital storytelling amenable to the web-based environment. Digital storytelling is an arts-based, participatory methodology wherein complex and compelling narratives are cocreated that elucidate the human dimensions of health and illness in ways that foster deep insight [16,17]. Additional aims of digital storytelling include empowering participants through personal reflection and growth, presenting experiential information for health promotion, informing public policy and advocacy, and providing multisensorial data to support public health research and evaluation [16-20].

An area of research that has received less attention is digital storytelling for knowledge translation [19]. The creation and addition of patient-perspective evidence to our website in the form of digital stories presented a critical opportunity to design and deliver innovative and engaging information that was not available elsewhere but that also has the potential to be easily shared with larger audiences. However, we did not know whether the digital storytelling codevelopment approach would be acceptable to story cocreators, hereafter referred to as storytellers, considering the private nature of painful sex, traumatic past experiences, and related embarrassment or shame. Furthermore, an understanding of emotions associated with this methodology in the context of endometriosis-associated painful sex was necessary for ensuring that participants were adequately supported and their experiences appropriately evaluated in subsequent research. Given the personal nature of these stories and the prominence of partners in storytellers’ experiences, knowledge of whether, with whom (ie, a partner, family, others with endometriosis, and the public), and through what means (ie, small groups, facilitated groups, private web-based groups, and publicly available websites) storytellers are willing to share their stories was considered crucial for determining knowledge dissemination strategies and target audiences in future initiatives.

### Objectives

The purpose of this study was to pilot-test a digital storytelling workshop focused on endometriosis-associated painful sex to understand storytellers’ experiences of workshop participation. We aimed to assess the feasibility of story cocreation and sharing and, more specifically, (1) the emotional impact of workshop participation, (2) the acceptability of a workshop for the subject matter, and (3) storytellers’ willingness to share their stories with broader audiences as a method for knowledge translation.

## Methods

### Design, Study Team, and Setting

We adopted a community-based participatory research approach incorporating the tenets of patient-oriented research and integrated knowledge translation, defined by a mutually respectful partnership between researchers and communities [21,22]. We defined a community in this context by disease membership. Throughout this pilot study, community partners included the Endometriosis Patient Research Advisory Board at the British Columbia Women’s Hospital, which supports 9 patient partners with endometriosis in guiding the research objectives of the University of British Columbia Endometriosis Pelvic Pain Laboratory. Community partners also included 6 storytellers who were active partners and coresearchers throughout the research process. In total, 67% (4/6) of these storytellers were also members of the patient advisory board. Clinician investigators contributed >20 years of experience in endometriosis clinical care and research. Researchers had qualitative methodological and health IT expertise. As a study team, we held an interdisciplinary philosophy of endometriosis care and research that considers the various components of the overall person with chronic pain rather than only the specific endometriosis lesions that indicate the disease itself.

### Ethics Approval

This research was conducted in British Columbia, Canada, beginning in the fall of 2020 during the COVID-19 pandemic, when public health orders and the curtailing of in-person research were in effect. Therefore, we conducted all activities on the web supported by Zoom (Zoom Video Communications). We obtained research ethics approval from the harmonized University of British Columbia and Children and Women’s Research Ethics Boards (H20-01969).

On the basis of honorarium restrictions from our research ethics board, we compensated the storytellers with CAD $170 (US $127.42) for the time and commitment to the study. Workshop participation alone exceeded 20 hours over 3 months, with additional time spent completing the reflective journals, participating in the focus group, and contributing to research meetings and discussions.

### Recruitment and Informed Consent

We recruited storytellers from a pool of people with lived experience of endometriosis-associated painful sex who had previous experience working with the laboratory or the British Columbia Women’s Hospital Foundation as patient partners.

We obtained consent at different study time points as there were multiple study components and we wanted to be respectful of the need for ongoing consent. Initially, this was consent to participate in the workshop, complete weekly reflective journals, and participate in a subsequent focus group. We considered the digital assets created to be the property of the storytellers. We sought consent to share their digital story after workshop completion with options to share privately with the research team; screen their story at a public event; or share widely, including but not limited to social media channels, newsletters, and selected websites for educational, research, knowledge translation, or advocacy activities. We also included the opportunity for storytellers to formally waive their right to confidentiality at the end of the study as storytellers were the owners of their stories and referring to them by a pseudonym, as is common in research, may unintentionally serve to strip them of the power to own their own experiences. Each of the 6 storytellers also reviewed, edited, and approved this manuscript before publication.

### Digital Storytelling Workshop Procedures

The storytellers created their digital stories during a web-based workshop consisting of five 2-hour sessions plus individual consultations spread over 6 weeks using a methodology developed and facilitated by The Center for Digital Storytelling in Berkeley, California, hereafter referred to as StoryCenter [23]. The storytellers attended the workshops and dedicated time between sessions to work on their stories, estimated by StoryCenter to be approximately 20 hours. This approach engaged storytellers in a group-based process to create and share narrative accounts of life events in a 3- to 5-minute video. An external collaborator with experience developing stories using the StoryCenter methodology and a clinical counselor experienced in sex therapy, endometriosis, and trauma-informed practice facilitated the workshops. StoryCenter facilitators were trained in a 7-step story development model established by Lambert [24] that outlines a framework for self-discovery that uncovers deeply rooted emotions regarding a pivotal moment in time (Textbox 1). The research team did not direct the workshop activities. However, the study co–principal investigator, who has >10 years of experience as an endometriosis specialist, was available to review the dialogue included in the stories for scientific accuracy at the storytellers’ request.

**The 7 steps of digital storytelling.**

- Step 1: owning your own insights
  Storytellers focus on creating a story that is unique to themselves and take an intimate glance at life events and how those events have changed them. This journey of self-discovery is meant to help people uncover the meaning in their story.
- Step 2: owning your emotions
  Storytellers are encouraged to get in touch with their emotions and connect what is meaningful to them with the emotions in their story. It is the emotions that will allow the story to resonate with others.
- Step 3: finding the moment
  Storytellers are asked to pinpoint a moment of change where their story had a turning point.
- Step 4: seeing your story
  Storytellers are encouraged to visualize their story through exercises that recall memories and explore the meaning of relevant images. They search through personal photographs or create artistic representations that will become the visual experience of their story.
- Step 5: hearing your story
  Expanding on visual recall, storytellers are encouraged to focus on audio, including voice-over recording, ambient sounds, or music while considering the role sound plays in evoking emotions, creating meaning, and contributing to the story arc.
- Step 6: assembling your story
  Storytellers review the script, images, and sounds associated with their story and start building the story in a digital environment. This includes real-time editing, sharing progress with the group, and implementing feedback. This may involve storyboarding and multiple iterations as the storyteller considers the content, images, sounds, length of the story, and intended silence.
- Step 7: sharing your story
  Storytellers revisit the initial conceptualization of their story, remind themselves of the purpose of their story, and reflect on the story’s relevance to their intended audience. This is when storytellers can share their digital stories with the group before making final revisions.

### Data Collection

We collected data from (1) reflective journals, (2) participant observations of the digital storytelling sessions, and (3) a focus group interview (Textbox 2).

**Description of the data collection methods.**

- Reflective journal
  The purpose of the journal was to capture storytellers’ thoughts, feelings, and experiences as they created their own stories. Each week, storytellers completed a journal entry on the REDCap (Research Electronic Data Capture; Vanderbilt University) electronic survey platform. Guiding free-text questions asked storytellers to briefly describe the focus of the week’s session, how the session was different from that of the previous week, what surprised them, and the emotions that were brought up.
- Participant observation
  A study co–principal investigator with previous experience creating a digital story through a StoryCenter workshop attended the digital storytelling sessions and recorded handwritten notes based on her observations of the storytelling process. The Endometriosis Patient Research Advisory Board advised that having an observer in the web-based room would likely be more acceptable to storytellers than sharing a verbatim recording with the research team because of the highly personal nature of the discussions.
- Focus group
  In total, 2 research team members conducted a focus group with the storytellers following workshop completion. The facilitated discussion centered on the thoughts and feelings experienced by the storytellers during the workshop sessions and their personal and collective journeys from story conception to completion. The focus group was audio recorded using Zoom videoconferencing software (Zoom Video Communications) and transcribed using the Temi automated service.

### Analytic Approach

We analyzed the reflective journals, observation data, and focus group data using a qualitative interpretive description approach, an inductive analytic approach designed to support applied qualitative studies and generate practical and clinical applications [25]. The qualitative data were housed and managed in NVivo (version 11; QSR International). In total, 2 study team members independently reviewed the reflective journals, observational notes, and focus group transcript to identify emergent ideas, patterns, and diversities that represented potential initial codes. We then deliberated and agreed on the preliminary inductive codes as the basis for a coding schedule, which we applied to the entire data set. Following this initial coding, we used constant comparative techniques to compare data within and across storytellers, moving from descriptive to more interpretive analysis [25]. This process enabled us to group and regroup the inductive codes into categories and from categories into main themes to address the study aims. While writing our findings in manuscript form, we reflected further on our interpretations of the data and refined the findings based on input from the larger study team. Interpretive description enabled us to draw on patient and clinician expertise throughout data collection and analysis and generate key considerations for a subsequent research plan.

## Results

### Overview

A total of 5 women and 1 nonbinary storyteller aged between 19 and 39 years consented to participate in this project. In total, 67% (4/6) of the storytellers identified as heterosexual, and 33% (2/6) identified as bisexual. Of the 6 storytellers, 5 (83%) identified as White individuals, and 1 (17%) identified as a Southeast Asian individual. Endometriosis had been a part of the storytellers’ life for 4 to 22 years. All storytellers (6/6, 100%) had experienced the hallmark symptoms of endometriosis, including painful sex, painful periods, and chronic pelvic pain. All had tried various treatments, including but not limited to medication, psychological therapies, surgery, physiotherapy, and various complementary alternative medicines. In total, 50% (3/6) of the storytellers had previously shared their endometriosis experiences publicly.

We characterized storytelling workshop participation and the feasibility of story cocreation by describing storytellers’ experiences of (1) opportunity, commitment, and connection; (2) complex emotions that were healing; and (3) a desire to share. We also outline key learnings to inform the logistics of future research.

### Opportunity, Commitment, and Connection

We informed the storytellers of what to expect from the workshop, but they were not required to prepare anything before attending the first session. However, we found that almost all storytellers arrived with a fully formed idea and subsequently drafted a script on day 1. It was as if these individuals had been waiting for an opportunity to speak openly and honestly about their experiences with endometriosis to others. When describing their desire to join the project, they spoke of an interest in the digital storytelling medium as a unique opportunity to learn a new form of technological communication. They described perceiving the use of an arts-based storytelling medium as enabling them to control the aspects of their story that were public to others and to stay anonymous by using abstract visual images if so chosen:

I thought the digital aspect of it was a really cool way to share this personal, intimate story, which, I mean, I ran a blog and I didn’t know how to talk about sex. I thought it was a great way to have this sort of discussion. You could share your story but not have to show your face as well. So, I thought that was a great option.

Storyteller 1

Even from the first session, the storytellers demonstrated tremendous commitment to the project and especially to each other. This commitment was apparent despite the multiple ways in which endometriosis disrupted their lives and the fact that the project was taking place during a global pandemic and local wildfires. For example, the researcher reflected on the second workshop in their written notes:

Each participant, as well as all facilitators, shared that they had had a challenging week. One participant had only left their house really yesterday because the [forest fire] smoke finally eased; 1 participant had a COVID scare and had been in quarantine for the week, another had a health scare and had been in hospital last night, another had a horrible time with the smoke and was having health issues, another was dealing with childcare issues. All participants indicated that they were feeling quite exhausted from the week, and I am astounded that they all still showed up for the workshop...Even more astounding was that they had all prepared a story to share.

This commitment continued throughout the project. The storytellers dedicated, at minimum, the 20 hours expected and continued to make modifications and adjustments to their story for approximately 3 months after the workshop sessions had ended. The process of connecting to share common life challenges beyond endometriosis appeared to be unifying and affirming. The group norms regarding commitment in seizing the opportunities for developing a digital story went beyond asset building to embrace connection among the storytellers. The bond that the storytellers developed with each other appeared to serve as ongoing motivation.

The storytellers commonly noted that they entered the workshop process feeling lonely. Their experiences with endometriosis often isolated them from friends and family who did not share the same experiences, and so several storytellers were looking to meet others in search of validation, community, and connection. Sharing this experience with other storytellers with whom they could relate and the nature of the facilitated story circles resulted in immediate camaraderie. The connections they built with each other throughout the sessions allowed them to connect as people, not just as people with endometriosis, as a storyteller shared:

In the beginning, I was really, really anxious about it, but even halfway through the first session, that anxiety definitely decreased just at how welcoming and accepting and just how common it felt our experience was. We had this like connection that was beyond just Endo, having Endo. It was really great.

Storyteller 2

Hearing honest accounts of the struggles that other storytellers had endured and the common threads that ran through their stories facilitated a deeper understanding and appreciation of their own and each other’s experiences. A storyteller commented the following:

I was very moved by [name] story as so much of my own is captured in her words. Again, I felt a sense of connection and of a deep understanding from my fellow participants, which was really beautiful and made me want to think about how to connect further with these women or others who have Endo.

Storyteller 3

Feeling seen, heard, and understood by others in the group validated the storytellers’ experiences and contributed to them feeling less socially isolated and alone. They conveyed hope in the collective, with a storyteller commenting on the “many shared experiences uniting all of us.” All storytellers (6/6, 100%) characterized the workshop as a supportive group and a safe, respectful space wherein they felt cared for. Support was offered by all storytellers in all sessions in the form of words of gratitude and encouragement when storytellers shared their experiences or stories and acknowledgment and acceptance of the emotions that arose. The following phrases were offered to fellow storytellers after they shared a draft of their story script: “beautiful,” “incredibly powerful and deeply vulnerable,” “writing is exquisite,” “speaking so openly,” and “I can relate to what you described.” Furthermore, as the workshop progressed, the connection with coparticipants grew into a sense of belonging, camaraderie, and community and a desire to continue supporting each other and others affected by endometriosis. Feasibility was demonstrated through 100% engagement in the workshops.

### Experiencing Complex Emotions That Were Healing

#### Overview

The storytellers described participation in the workshops as a complex emotional experience that included intense vulnerability stemming from excitement and commitment to the research but coupled with fear and anxiety. The storytellers expressed fear, anxiety, and worry about who else would be in the workshop before it began but also because they were asked to reveal personal and private details. They were initially apprehensive about unpacking their emotional traumas with others, fearing the personal toll it might take and unsure of how fellow storytellers would react. However, the storytellers viewed the workshops as a form of advocacy that might help others with endometriosis. This commitment to the self and others appeared to motivate them to be involved despite their initial fear and anxiety. A storyteller stated the following:

It definitely felt raw and vulnerable, of course, to talk about the painful sex aspect and simultaneously felt super important because this is such a stigmatized aspect of endo that I think is just so important to talk about and to raise awareness over.

Storyteller 6

The storytellers came to the workshop with diverse experiences of endometriosis and therapy; however, all had experienced some form of related trauma that affected their lives in multiple ways. The digital story creation process involved recalling details of past difficulties and traumas, which were deeply emotional, as described by a storyteller:

I had a very emotional week with getting the video together as it took me back to that time...My story just made me really sad that I now will never be able to have my own kids even though I had decided to make that decision years ago. I haven’t felt myself be this emotional about not being a mom, about how my life has changed, for a few years now. I felt I had been doing well up until a few months ago, but this stirred up old emotions which I thought I had moved past and was ok with. But I think when you wanted something so bad, you accept it didn’t happen, but it can still hurt.

Storyteller 5

The sharing of such personal and private stories was particularly emotional, evoking feelings of sadness, anger, and grief, which came as a surprise and was described as hitting some storytellers harder than they had expected. Hearing and viewing the stories shared by the other storytellers also evoked strong emotions of sadness, anger, and grief. Although these reaffirmed the storytellers’ own experiences, they also highlighted the difficulties that people with endometriosis often experience for years. A participant journaled the following:

Actually, hearing that others genuinely have the same experiences as me. This session was sad and devastating. It was hard to hear how much Endo and pelvic pain had negatively affected their lives.

Storyteller 3

As the storytellers progressed through the 6 weeks of workshops, they reflected on the ongoing emotional work involved and the toll this took on their “physical and mental self,” as summarized by a storyteller’s journal statement: “man, am I ever wiped out!”

#### Emotional Healing

As the storytellers were guided through the 7-step story development model, they were continually encouraged to support each other; note what they wanted to hear more about; and use validating, reflective, and supportive language. The authenticity with which these words were spoken, the visual and auditory exercises, and the sharing of a common experience seemed to contribute to the storytellers’ emotional healing and personal empowerment. The workshops provided an opportunity for some storytellers to engage in emotional healing from the trauma associated with having a long-term chronic pain condition. During the focus group, a storyteller reflected the following:

I just sort of felt like this was this unique opportunity to actually, instead of doing like a writing version of my story to do this visual version, which actually just felt super serendipitous and kind of, for me, actually, it felt like it sort of completed this healing process.

Storyteller 4

This storyteller pointed to the power of the visual testimonial to assist in believing what is seen and sharing what is felt. Furthermore, the storytellers’ authentic, raw, and vulnerable digital stories and the work of creating them could be seen in the third person such that they could view their story anew and with compassion.

In addition to the group workshop sessions, all storytellers worked on their story scripts, audio, and visuals on their own time, which involved activities such as writing, reading their past journal or diary, locating and viewing old photographs and cards, and painting or creating art. These activities were an opportunity for storytellers to take time to revisit and reflect on old memories. Although some memories were emotionally painful, this work of vulnerability and revisiting losses seemed healing rather than inciting reinjury. Through continued commitment to the story creation process and staying present with complex emotions, the storytellers were able to place some of these memories and experiences in the larger context of their lives and appreciate the challenges they had endured and the gains they had made. The storytellers reflected on the emotional but ultimately healing process of revisiting their own stories, reading their scripts aloud, hearing their voices, and seeing the finished product:

I felt when reading the script itself, it took me back to that time, and I couldn’t believe what I went through. I was also having a rough week with my external world and comments being made about how life isn’t life if you don’t have kids. This, of course, made me emotional, so I thought reading and revising my script would be difficult, but instead of feeling weak and unworthy, I felt strong and brave thinking of all I have overcome.

Storyteller 3

The storytellers also noted that the journey highlighted their strengths and challenges and illuminated their personal relationships with control and perfectionism. However, for some, the opportunity to share their videos had a cathartic effect of letting go:

For me, it has been a journey to get to know myself better...how I want it to be perfect, or wanted to show many aspects, and I have to let it go.

Storyteller 5

Feeling accountable to workshop coparticipants and providing a script, audio, visuals, and ultimately, a final video appeared to inspire the storytellers to continue to revisit and work through difficult emotions.

The inclusion of 6 storytellers was considered the ideal size, allowing for authentic sharing and supporting of others in the group. A couple of the storytellers (2/6, 33%) indicated that the workshop complemented the “inner work” or counseling they were already engaged in. Furthermore, the storytellers considered it vital to work with an experienced facilitator who had training in trauma-informed practices and could guide them through the story creation process and assist in developing their ideas. The availability of the facilitator outside the workshop sessions was a noted strength and helped people move their stories toward completion. Although a counselor was present during the workshop sessions and a couple of the storytellers participated in individual sessions with the counselor outside the workshop, the storytellers expressed a desire for 1:1 clinical counseling support throughout the workshop process to help manage the intense emotions that arose. Interestingly, some storytellers (2/6, 33%) recommended that the weekly reflective journals, which we had included to collect data, be integrated into StoryCenter workshop approaches in subsequent research as the reflective questions helped them develop their stories and cope with their emotions.

#### Personal Empowerment

The group configuration of the workshops, wherein the storytellers described a deep connection with others and emotional healing, ultimately served to enhance the storytellers’ personal empowerment in their journey to create their videos. The storytellers appreciated coming to learn from each other about the various ways in which endometriosis affected their lives and also how each coparticipant had managed in the face of tremendous challenges. Bearing witness to vulnerable emotions seemed to ignite unity and a normative drive to tell it like it really is. Affirmation and validation from being believed by fellow storytellers, as well as empathy and connection with others’ stories, were prevailing emotions. A storyteller’s comments exemplified the sense of personal strength she drew from hearing and seeing collective struggles and connecting with the other storytellers:

Even though all our experiences with Endo are nuanced, there are many common themes—validation with diagnosis, importance of support networks, feelings of loss, hope even amidst great struggle. We are stronger than we may sometimes feel.

Storyteller 3

The storytellers were proud of each other and of coming together to support each other throughout the story-making process. They described drawing strength from their fellow storytellers, in whom they witnessed “so much courage and bravery.” The storytellers’ commentaries conveyed their sense of pride from persevering through the project despite some difficulties and accomplishing a personally meaningful goal, as evident in the following journal entry:

It has taken me more time and effort to sit down and work on it. Today I worked on the video, and it felt good. I am happy with me. The outcome and the video feel very much like me.

Storyteller 2

For a couple of the storytellers (2/6, 33%), the sense of connection to self, authentic representation, and personal empowerment gained from the workshop extended to other aspects of their lives, including the making of important life decisions. A storyteller further described the creation of her digital story as a process of self-actualization that was helping her feel empowered to trust and listen to her body as a way to cope with endometriosis, something she indicated she had spent a lifetime resisting:

What this video did for me was helped me to recognize that I have been doing so much work on listening to my body and what my body is actually telling me. And sometimes, I get a little left-brain where I’m over-analyzing and thinking. And so, when I pull myself back into my body, there’s a pretty clear message that like, [something] just doesn’t feel right.

Storyteller 6

For this storyteller, her intuition was perhaps the pivot and product for introspective work. Overall, all the storytellers (6/6, 100%) characterized the workshop and the creation of their digital stories as empowering.

### Opening Up About Painful Sex

All the storytellers (6/6, 100%) described coping with the hidden symptoms of endometriosis for years alone. Considering the sensitive and personal nature of gynecological disease and the stigma of sexual ill health, some (2/6, 33%) had few opportunities to talk with others and kept their thoughts to themselves. Other storytellers (4/6, 67%) had previously shared their stories of endometriosis, 3 of whom had shared their stories publicly. However, for all, there appeared to be a transition from private thoughts to comfort in talking openly about endometriosis generally and painful sex specifically.

For some, the added importance of painful sex as a subject matter affirmed their testimonies as audience worthy, and the format of the workshop helped them overcome previously internalized barriers to discussing this typically private and stigmatized topic. Others weaved their life contexts into their endometriosis, layering their painful sex narratives more broadly:

I found that I couldn’t really think about a story specifically relating to painful sex because it was just like my life...for me, it felt more natural to kind of give a bigger overview of my story and then incorporate just a little bit about painful sex.

Storyteller 6

As a personal topic that is often challenging to discuss, some storytellers (3/6, 50%) mentioned that their closest friends were not even aware of their endometriosis-related challenges and certainly not in the context of painful sex. The storyteller journals and workshop observations suggested that the act of isolating the effects of endometriosis to an internalized dialogue was all too common. A participant remarked on the dehumanization and desensitization that people who experience chronic health conditions might feel. They described feeling disconnected from the medical narratives created by and large by health care providers and appreciated being encouraged to speak honestly about a specific sensitive subject in a manner that was personally meaningful:

So much of our lives are open to people, to doctors, to nurses like how many people have seen me naked. It’s ridiculous! So sharing my personal story like this, it doesn’t really feel that personal anymore...to be encouraged and have a platform like this...we want you to be honest. It was very refreshing.

Storyteller 4

Despite feeling able to open up about painful sex in the workshop and create their digital story, a couple of the storytellers (2/6, 33%) also expressed concerns. Considering that sexual experiences often involve a sexual partner, these storytellers were compelled to assess the impact of sharing intimate details related to their partners in their stories:

My husband’s family is very conservative...so, I can definitely see there being some major ramifications for him, even though he says he would be fine.

Storyteller 1

Throughout the process, some of the storytellers (4/6, 67%) consulted with their partners and received their support. Others (2/6, 33%) opted to focus less on specific personal, painful sexual experiences and more on how endometriosis had affected their relationships.

After the workshop, all storytellers (6/6, 100%) consented to sharing their stories widely, including but not limited to social media channels, newsletters, and selected websites for educational, research, knowledge translation, or advocacy activities. We have now included the digital stories on our website, *Sex, Pain & Endometriosis* [15]. Interestingly, a storyteller initially did not consent as she wanted to keep her story private. However, she contacted the research team 6 months later, enthusiastic about sharing her story. She described the process of deciding as a lengthy deliberation with close friends and family wherein she weighed the potential personal and professional consequences of releasing her story against her desire to engage in advocacy.

The storytellers also conveyed their journey through the workshop as facilitating their emotional healing and their desire to share their stories as a form of letting go. They felt strongly that viewing the stories could help others who have endometriosis feel less alone, validate their challenges, and build a community. As such, 1 year following workshop initiation, we supported our storytellers in hosting a Facebook live web-based story screening and panel discussion event. Screening to the larger public was considered essential to try to reach people with endometriosis who might have more limited access to support and services, raise awareness of endometriosis-related struggles, and combat feelings of social isolation and loneliness. Furthermore, the opportunity to provide commentary through a panel discussion was deemed an essential complement to the digital stories. Overall, we found that the feeling of empowerment and desire for advocacy that arose from the workshops extended well beyond workshop completion.

### Key Considerations for Future Research: Preparation and Logistics

On the basis of the piloting of our digital storytelling workshop, we reflected on the logistical aspects that influenced the feasibility of project participation. We identified 6 key learnings to guide future research development and execution (Table 1).

**Table 1:** Preparation and logistic reflections and learnings.

Item	Reflection	Learning
Respectful design	We conducted five 2-hour sessions over 6 weeks with 6 storytellers. We received feedback that this was the ideal size, and support provided by the facilitator was just right. However, 5 out of 6 storytellers shared that the pace was too fast considering the complex emotional experience of story creation and the demands of their lives. In addition, the facilitator continued to work with storytellers on minor adjustments for months after workshop completion.	Flexibility should be built into the timeline out of respect for emotional coping and competing life events and to allow for finishing touches. There should be a good fit between the facilitator and the storytellers. Engaging with the facilitator on several occasions before the workshop helped create a shared vision, address potential sensitivities, and craft an appropriate approach for our context.
Accessibility	Digital storytelling requires access to a camera and a computer and a basic level of technological ability. Despite communicating the needs of the project during the informed consent process, some storytellers were surprised that they required access to technology and that it would not be provided.	Research teams should be extremely clear of the technological requirements and budget for laptops, phones, or tablets to ensure inclusion of those who do not have access to suitable technology.
Convening on the web	All activities took place remotely through Zoom videoconferencing software (Zoom Video Communications), phone, or email. Despite this, we found the web-based environment to be surprisingly intimate.	Although the technological accessibility of each population should be considered, overall, the web-based format was acceptable for researchers and storytellers.
Supporting success	We found that storytellers revealed struggles in their journal entries that they did not reveal face to face. By monitoring journal entries, we were able to offer additional support that proved to be highly appreciated.	Monitoring of journal entries on a regular basis can alert the study team to issues arising for storytellers to address challenges and put in place supports.
Reducing harm	The storytellers were offered a 1:1 session with a clinical counselor to address difficult emotions that arose during the storytelling process, or they could be referred to targeted support for longer-term issues. The storytellers indicated a desire for additional counseling sessions.	Future projects could consider the resources available to support storytellers after the workshop or have a list of potential local counseling services.
Valuing storyteller contributions	We were severely limited by our research ethics board in the financial compensation we could provide despite >20 hours of work being required of storytellers.	Teams should explore alternate ways of financially supporting storytellers that still comply with local ethics boards.

## Discussion

### Principal Findings

This study is the first to pilot-test digital storytelling with people who experience endometriosis generally and painful sex specifically. Overall, we found that story cocreation and sharing were feasible and acceptable based on storytellers’ experiences of the workshop as an opportunity to which they demonstrated considerable commitment and in which they found connection; a way to engender complex emotions but that ultimately led to emotional healing and personal empowerment; and a way to open up and talk about painful sex and, ultimately, a desire to share. Furthermore, all the storytellers (6/6, 100%) consented to sharing their digital stories widely and went on to host a web-based public screening and panel discussion. Although feasible and acceptable, the findings and logistic considerations that arose during the project allowed us to identify key learnings for future research.

It is well documented in the chronic pain and endometriosis literature that people feel silenced, dismissed, disregarded, and even actively discouraged from sharing their experiences with others [8]. The observation that storytellers came prepared with a story to the first workshop session speaks to the need for opportunities to authentically narrate illness struggles and to be seen and heard. Furthermore, the storytellers expressed feelings of validation and recognized the potential benefit to others beyond the workshop participants. This suggests the value of opportunities to validate their experiences and the need to complement biomedical explanations and accounts of endometriosis with experiential narratives. Access to the experiential accounts of peers can provide insights, support, and hope, with research highlighting patients’ appreciation of the experiential knowledge offered through digital storytelling [19].

Research has documented a considerable delay in endometriosis diagnosis that has in part been attributed to lack of awareness, normalization of symptoms, and reluctance among health care providers and those with endometriosis to discuss symptoms. Focusing on menstrual irregularities, a primary symptom of endometriosis, Seear [26] drew on work by Goffman to argue that menstruation is a “discrediting attribute” and that disclosure renders people vulnerable to stigmatization. Participants in the study by Seear [26] experienced ostracism, rejection, criticism, invalidation, and trivialization from family, intimate partners, health care providers, and employers when they disclosed menstrual abnormalities. This led the participants to mask and strategically conceal menstrual symptoms. Painful sex can be viewed as another “discrediting attribute”—one that, even in the context of diagnosed endometriosis, is difficult to discuss, concealed and often delinked from the disease and its management. Although both self-stigma and social stigma might contribute to diagnostic delay, they might also catalyze and sustain diminished psychosocial well-being [6].

Previous research suggests that digital storytelling is appropriate for populations that face high levels of stigma, such as sexuality and reproduction, chronic disease, intimate partner and gender-based violence, mental health, and substance use disorders [16,18,27-30]. For example, early research with veterans and firefighters with posttraumatic stress disorder suggests that digital storytelling can challenge stigma and promote mental health treatment seeking [31-33]. In our study, the storytellers positioned digital storytelling as a possible means of validating their endometriosis struggles, and we posit that it might also combat the social and internalized stigma of endometriosis and painful sex.

Furthermore, as an integrated knowledge translation strategy, the viewing of authentic and relatable personal experiences of peers via digital stories can perhaps challenge misconceptions about endometriosis symptoms as normal, disrupt societal stereotypes that perpetuate stigma and shame, help people recognize painful sex as a symptom of endometriosis, enhance confidence to self-advocate and seek treatment, and combat stigma. These stories may further enhance empathy among family members, friends, and intimate partners as a tailored knowledge translation tool. Similarly, digital storytelling can potentially sensitize health care providers to appreciate and anticipate the diverse lived illness experiences [16] and normalize discussions about endometriosis symptoms. As ours was a pilot study, we did not assess the impact of the digital stories on people outside the workshop; however, we now know that it is feasible to share these stories with broader audiences. Future research on the impact of doing so will be an essential next step.

The findings from this study suggest that the method of digital storytelling lends itself well as a therapeutic application, which may also be a pressing need for people with endometriosis. Our findings align with the work of Fiddian-Green et al [17], who developed a conceptual model of how the group-based *process* of digital storytelling (as developed by StoryCenter) as an applied health intervention can contribute to increased socioemotional well-being. They posit that the group process of creating, sharing, and discussing a digital story can result in emotional acceptance, social support, and self-efficacy that can enable individuals to engage in positive health behaviors. Indeed, the storytellers in our study reported emotional acceptance, social support, and self-efficacy and lend credence to the proposed model. An unexpected finding was how much the workshop process led people, some of whom had struggled with endometriosis for 22 years, to new insights about themselves and the condition. It also served to empower storytellers in owning and viewing their own stories and engender the desire to support the journey of others. Viewing our findings in the context of the therapeutic benefits of group psychotherapy [34] provides some insights. From this perspective, group work is the most important and effective factor in promoting healing and change, wherein group validation is a powerful tool. Furthermore, it seemed that a good number of the therapeutic factors of group psychotherapy were satisfied through digital storytelling workshop participation, such as bearing witness and being witnessed, instillation of hope, altruism, interpersonal learning, group cohesiveness, and catharsis.

We found a therapeutic benefit despite the group process in our study consisting of web-based sessions stretched over 6 weeks rather than the typical 3-day in-person workshop. This suggests that alternative modes of workshop delivery are perhaps equally effective and more accessible for some. Moreover, the digital storytelling public screening and panel discussion as a form of knowledge translation also appeared to function as a therapeutic intervention itself and perhaps extend the proposed model by Fiddian-Green et al [17]. Future research evaluating the therapeutic value of the process of digital storytelling as well as public sharing of created stories is warranted. Similarly, it remains to be seen whether digital storytelling can be woven into clinical practice as a psychosocial therapeutic aid and an area ripe for research.

Of note, the timing of our pilot study corresponded with the early phase of the COVID-19 pandemic, and web-based workshop delivery was necessary. The opportunity to come together at a time when anxieties were high perhaps helped the storytellers forge connections with each other. Methodologically, the switch to web-based engagement as well as the gathering of web-based qualitative data were feasible and certainly afforded benefits in terms of ease of storyteller participation, ease of data collection, and reduced financial costs. The benefits of using the web-based platform Zoom in qualitative research during the COVID-19 pandemic have included the therapeutic value and data richness afforded by interviewing participants who were able to stay in their own home and the reduced costs to extend recruitment reach and inclusivity [35]. Considering these benefits, it is highly likely that web-based research is here to stay in some machination, although this might change some aspects of participatory methods and what engagement entails. Our research afforded early insights into web-based engagement in participatory research, and feasibility was demonstrated by 100% engagement. However, attention to the ways in which web-based research participation might create other risks or challenges, such as lack of privacy or a safe space in which to engage in web-based communication, is warranted.

### Limitations

Despite our findings that the web-based digital storytelling workshop was feasible and acceptable to storytellers, study limitations are important to consider. First, we were cautious and calculated in our recruitment efforts as we wanted to minimize the potential for harm to storytellers. We introduced the study to people we thought would be open to workshop participation, were connected to an existing support system, and were further along in their health care journey (had endometriosis-specific care). This approach allowed us to connect with storytellers outside the workshop to find solutions to the challenges that emerged. It remains unknown whether a research team could respond appropriately and whether a storyteller would be receptive to this type of engagement in future studies with more broad recruitment approaches. Although this remains unknown, we identified important mechanisms to prevent challenges from occurring and systematically monitored for challenges throughout the sessions. A second study limitation was the limited variability in some of the storyteller characteristics, particularly regarding being well informed about endometriosis and having current access to endometriosis care. Likely, the storytellers did not represent the full diversity of experiences of endometriosis or painful sex. For example, none of the stories focused on treatment successes. Ways of recruiting storytellers with diverse experiences would ensure that a broader range of experiences are represented in future research. However, perhaps grouping storytellers according to important experiences or characteristics would be appropriate considering that the homogeneity of storytellers in our study appeared to facilitate group cohesion with a therapeutic benefit. If bringing storytellers together anew, and considering the emotional work involved, targeted efforts to create a safe space up front would be necessary. Third, by creating bonds between people searching for a community, we inadvertently created a space for this community to exist. We did not have a mechanism for the storytellers to stay connected upon study completion. Acceptable means of opting in or out of connecting with fellow storytellers after the research remain to be determined.

### Conclusions

There is evidence from this pilot test suggesting that a web-based digital storytelling workshop related to endometriosis is feasible and acceptable for storytellers. As a knowledge translation tool, our approach holds promise for raising awareness and validating illness experiences and, of equal importance, for combating social and internalized stigma. The therapeutic benefit of this workshop warrants future research as a brief, accessible, and possible complement to existing clinical and psychological supports. Furthermore, digital storytelling could be a brave new frontier capable of reaching ever-increasing geographies through web-based workshops and the norming of streaming events and digital products on the web.

## Data Availability

The data sets generated and analyzed during this study are not publicly available as participants did not consent to making their data publicly available, but they are available from the corresponding author upon reasonable request.
